# The effects of exposure to objective coherence on perceived meaning in life: a preregistered direct replication of Heintzelman, Trent & King (2013)

**DOI:** 10.1098/rsos.160431

**Published:** 2016-11-23

**Authors:** Kaylin Ratner, Anthony L. Burrow, Felix Thoemmes

**Affiliations:** Department of Human Development, Cornell University, G06 Martha Van Rensselaer Hall, Ithaca, NY 14853-4401, USA

**Keywords:** meaning in life, coherence, well-being, replication

## Abstract

Having a sense of meaning in life (MIL) has been acknowledged as a catalyst to psychological flourishing. As such, understanding ways to promote MIL represents a worthy goal for those interested in bolstering positive outcomes. This study sought to replicate the findings of Heintzelman, Trent & King (2013 *Psychol. Sci.* **24**, 991–998 (doi:10.1177/0956797612465878)), who found that MIL could be influenced by external stimulation. Their findings suggest that exposure to coherent stimuli produces significantly higher MIL scores than exposure to incoherent stimuli. Using materials and methodology provided by the corresponding author of the original paper, this study attempted to directly test this manipulation under conditions with increased statistical power. All tests, however, failed to replicate. Possible explanations for these discrepant findings are discussed, and potential future directions for this area of the literature are proposed.

## 1. Introduction

Since Viktor Frankl's landmark memoir, *Man's search for meaning* [1], finding meaning in life (MIL) has been cited as an integral part of psychological health and flourishing. To date, scientific evidence has largely been united on the advantages of being able to sense MIL, or the feeling that one's life is coherent [2] and/or significant [3]. Among the many positive outcomes delineated, increased psychological well-being [4], heightened happiness and life satisfaction [5], improved interpersonal functioning [6], a greater proactive health orientation [7], increased longevity [8], and decreased existential anxiety [9] and psychopathology [10] have been noted. Given the abundance of empirical evidence supporting the benefits derived from sensing MIL, understanding the sources that give rise to this sense represents a particularly important issue.

Among the most recent forays into what may contribute to or attenuate perceptions of MIL is a provocative set of experiments conducted by Heintzelman, Trent & King [11]. In their four-study paper, the authors tested if self-reported MIL could be influenced by external cues. Recognizable patterns in one's life, significant connections, and the degree to which events and ideologies seem consonant with one another have been theorized to lend themselves to perceived meaningfulness (e.g. [2,3,12,13]). As Heintzelman *et al*. [11] put forth, detecting patterns in the environment is an adaptive process for both humans and animals [14] and meaning is susceptible to experimental manipulation (e.g. [15]). To test whether external stimulation could lead to changes in perceived MIL, participants across Heintzelman *et al*.'s studies were randomly split into coherent and incoherent groups. In the first two studies, individuals in the coherent condition saw 16 unique photos of trees in seasonal change presented in either a repeating natural (spring, summer, autumn, winter) or unnatural (e.g. autumn, spring, winter, summer) pattern. In the incoherent condition, individuals saw the same 16 tree photos, but in a randomly presented order. In the same paper, Heintzelman *et al*. [11] performed two additional studies in which individuals were randomly assigned to either a coherent or incoherent condition, but instead of pictures of trees, participants were presented with word triads. In the coherent condition, participants were exposed to word triads (cf. [16]) with a common remote associate (e.g. ‘playing, credit, report’ (remote associate: card)). Participants in the incoherent condition were instead exposed to discordant word triads (e.g. ‘playing, plush, care’). In each of these four studies, participants exposed to coherent stimuli reported higher perceived MIL scores than those exposed to incoherent stimuli.

As interpreted by Heintzelman *et al*. [11], the findings of their study may represent a reflection of detected meaning in the environment given humans' natural proclivity to search for pattern and perceive significance [14,17]. Should this effect exist, it would have tremendous theoretical implications, especially for those interested in understanding what factors influence this unequivocally beneficial sense. Although still a very distal goal, taking these first steps and understanding the mechanisms of how the environment influences perceived meaning may eventually inform the ways we try to help vulnerable groups (e.g. [18]) detect a reliable sense of MIL. It is interesting to note, however, that the findings of Heintzelman *et al*. [11] are somewhat difficult to fully reconcile with some existing meaning-related theories.

According to Heintzelman *et al*. [11], the meaning maintenance model (MMM; [17]) might account for why this exposure–response relationship may be occurring. In experiments testing the MMM, the imposition of meaning onto alternative sources tends to occur following exposure to violations in expectancy. When such violations occur, it is thought that individuals may experience a threat to meaning which prompts them to then affirm meaning in readily accessible areas which remain intact—a term Heine *et al*. [17] call ‘fluid compensation’. In short, projecting meaning onto other areas in response to a threat to meaning is a reactive and restorative process which people enact when confronted with stories or stimuli which do not conform to expectation. Until Heintzelman *et al*. [11], no studies have explicitly measured subjective reports of MIL following exposure to a potential meaning threat in traditional MMM fashion (i.e. following exposure to a violation in expectancy). In some ways, however, the findings of Heintzelman *et al*. [11] run counter to the MMM. As noted by the authors, one might reasonably expect for meaning to be *promoted* in incongruent conditions following a violation in expectancy if one adheres to the MMM premise of fluid compensation. Although some questions surround its mechanics, it is clear that the findings of Heintzelman *et al*. [11]—by simply showing that perceived MIL can be manipulated by external exposure to coherence or incoherence—usher in a number of opportunities for future research. Such findings, no matter the directionality, could have a number of implications for understanding perceived MIL and its antecedents as a whole. These forthcoming pursuits, however, ultimately rest on the reliability of the demonstrated effect.

## 2. This study: direct replications with original materials

The objective of this study is to replicate Heintzelman *et al*. [11] with heightened statistical power in an attempt to explore the strength and reliability of this effect of exposure to objective coherence on perceived MIL. The interest in direct replication was born from three prior unregistered attempts at conceptual replication by the first two authors of this article. Each attempt varied slightly in terms of methodology, but all attempts used Heintzelman *et al*.'s Study 1 (seasonal trees) foundation and all yielded findings which suggested that MIL scores did not significantly vary between coherent and incoherent groups. Because some variations in the approach (e.g. differences in the tree photos used, pre-intervention questionnaires, covariates) might have given rise to the discrepant outcomes, a proposal for a direct replication was initiated. To prepare for replication, *post hoc* analyses of the original Heintzelman *et al*. [11] paper were conducted. An observed power of around 50% for three of the four reported studies was found. An analysis of the distribution of the four observed *p*-values for the focal hypothesis (*p*-curve; [19]) suggested that the distribution of observed *p*-values was not significantly skewed to the right, as would be expected when studying a false null hypothesis (*z* = 0.87, *p* = 0.81), thus, providing another argument for the worthwhileness of a direct replication endeavour.

A direct replication for Study 2 of Heintzelman *et al*. [11] is contained below. Study 2 was chosen for replication rather than Study 1 due to their similar nature: Study 1 is inherently contained in Study 2, and additional insights are offered by the presence of the ‘arbitrary’ (the unnaturally occurring, repeating pattern) condition alongside the seasonal coherent and random photo conditions from Study 1. In an effort to examine possible differences between tree and linguistic stimuli in eliciting changes in perceived MIL following exposure, an attempt to replicate Study 4 of Heintzelman *et al*.'s paper is also included. Study 4 was chosen for replication rather than Study 3 because of the modifications implemented in Study 4 to rule out possible confounds introduced by the procedures of Study 3. Added control in Study 4 is offered by using the same words presented in either coherent or incoherent orders accordingly across conditions and maintaining a fixed, experimenter-managed exposure time to each stimulus.

## 3. Method

### 3.1. Power analyses and participants

While a power analysis is always an important part of study design, it is especially important for replication studies to avoid under-powered replications. When conducting power analyses, it is typical to use the observed effect size of the unadjusted effect of the original study as the best guess of the population effect size. However, published effects can potentially be victim to overestimation [20]. To account for this, all power analyses assumed a population effect size that was 10% smaller than the reported effect sizes in the original Heintzelman *et al*. [11] paper. For the replication studies, sample sizes yielding at least 90% power were sought.

#### 3.1.1. Study 2

Study 2 of the original Heintzelman *et al*. [11] paper (*n* = 137) reported an effect size of *d* = 0.37 for the critical comparison of seasonal and arbitrary versus the random ordering. This analysis was conducted using a contrast code and the observed (*post hoc*) power of this comparison is 0.57. A power analysis (performed in GPower-3; [21]) for a comparison of two groups with unequal allocation (1 : 2 because individuals were randomly assigned to all three conditions; however, the contrast aggregated two groups together) with a 10% attenuated effect size of *d* = 0.33 yields a required sample size of 438 to achieve 90% power. To account for the fact that some participants may meet pre-specified exclusion criteria (outlined below), the pre-planned sample size was increased by 10%, for total proposed sample size of 482.

A total of 482 participants were successfully recruited to take part in our study from Amazon's Mechanical Turk (MTurk). For Study 2, participants' ages ranged from a reported 18 to 76 years (*M* = 34.86, s.d. = 12.26). Distribution of gender was roughly balanced with 259 reported females (53.7% of the total sample) and 220 reported males (45.6% of the total sample). To note, two respondents answered ‘other’ with regard to gender (0.4% of the total sample), and one respondent chose not to report a gender selection at all (0.2% of the total sample).

#### 3.1.2. Study 4

Study 4 of Heintzelman *et al*.'s [11] paper (*n* = 169) reported an effect size of *d* = 0.44 for the critical comparison between the incoherent and coherent group with an observed (*post hoc*) power of 0.81. A power analysis with a 10% attenuated effect size of *d* = 0.40 yields a required sample size of 266 to achieve 90% power. To again account for the possibility that some participants may meet the pre-specified exclusion criteria, the pre-planned sample size was increase by 10%, thus, bringing the proposed total sample size to 293 individuals.

A total of 294 individuals were successfully recruited to take part in our study from MTurk. The participants' reported ages ranged from 18 to 79 years (*M* = 34.73, s.d. = 11.4). This sample's gender split was, again, roughly balanced with 166 respondents reporting being female (56.5% of the total sample), and 128 reporting being male (43.5% of the total sample).

### 3.2. Materials

Original materials (tree photographs; presentation orders of the photographs for the seasonal, arbitrary and random conditions; colour swatches; and photographs for the coherent and incoherent word triads) were obtained from SJ Heintzelman (9 December 2015 to 19 April 2016, personal communication), corresponding author of Heintzelman *et al*. [11]. All response anchors and adaptations of existing measures will be performed identically to that which was described in the original paper and/or confirmed by Heintzelman in the personal communication. Although some of the following materials will not be used in the replications' analyses (e.g. contrast assessment, pattern check), these instruments were nonetheless included in the studies' design due to the desire to reproduce Heintzelman *et al*.'s original studies precisely.

#### 3.2.1 Tree photographs

Every participant completing the replication of Heintzelman *et al*.'s [11] Study 2 viewed a series of photographs of trees in seasonal change. Every tree photograph included at least one indication of season (e.g. flowers, snow) and had no other stimuli present in the photo (e.g. no people, paths, roads, artificial structures). Participants were also asked to rate the contrast of each photo on a scale from ‘Low’ (1) to ‘High’ (7) in addition to identifying the most frequently occurring colour in the photo from a presented array of colour swatches.

#### 3.2.2. Pattern check

At the end of the replication of Heintzelman *et al*.'s [11] Study 2, participants were asked if they would like to comment on anything they may have noted about the photos they viewed in the survey. For this optional response, participants were given a free response box with no time or character limit.

#### 3.2.3. Time spent on each tree photograph

Included in the Qualtrics survey software provided by the present authors' institution was timing software. The amount of time each participant spent on each tree photo was recorded. Participants were unaware of this recording. Time (recorded and reported in seconds) was summed across the 16 photos to create a total time score for each participant.

#### 3.2.4. Implicit positive and negative affect

Implicit positive and negative affect (IPA and INA, respectively) in the replication of Heintzelman *et al*.'s [11] Study 2 were assessed using five selected words from the Implicit Positive and Negative Affect Test [22]. The artificial words selected are ‘SAFME’, ‘VIKES’, ‘TUNBA’, ‘TALEP’ and ‘BELNI’. Each participant was asked to rate the extent each word sounds either positive (happy, cheerful, pleased) or negative (sad, worried, upset) in nature on a scale ranging from ‘Not at All’ (1) to ‘Extremely Much’ (7).

#### 3.2.5. Explicit positive and negative affect (EPA and ENA)

For both the replication of Heintzelman *et al*.'s Study 2 and Study 4, participants were asked to rate the extent to which they felt a selection of emotions both positive (happy, cheerful, pleased) and negative (anxious, worried) in nature. Participants rated these emotions based on the extent to which they felt the given emotion at the present time on a scale ranging from ‘Not at All’ (1) to ‘Extremely Much’ (7).

#### 3.2.6. Meaning in life

Across both replications, perceived MIL was assessed on scales which Heintzelman *et al*. [11] noted to be correlated at *r* = 0.90. MIL was rated on a scale ranging from ‘Not at All’ (1) to ‘Extremely Much’ (7) in both studies. In the replication of Study 2, MIL was assessed using items pulled from existing MIL measures: ‘I have found a really significant meaning in my life’ and ‘I have a sense of direction and purpose in life’ [23]; ‘My existence is very purposeful and meaningful’ and ‘As I view the world in relation to my life, the world fits meaningfully with my life’ [24] and ‘My life has a clear sense of purpose’ (Meaning in Life Questionnaire (MLQ); [25]). In the replication of Study 4, the MLQ Presence of Meaning subscale (reported Cronbach's *α* = 0.86; [25]) was used to measure MIL.

#### 3.2.7. Linguistic triads

For the replication of Heintzelman *et al*.'s Study 4, linguistic triads originally from Hicks *et al*. [16] were obtained. A series of 10 linguistic triads (a total of 30 words) were displayed to each participant. Coherent linguistic triads each had a unique remote associate (e.g. ‘coin, quick, spoon’ (remote associate: silver)) which was not explicitly divulged to the participants. Incoherent triads, created for the purposes of Heintzelman *et al*. [11] using the same words as those appearing in the coherent triads but in new combinations, did not have a distinguishable remote associate (e.g. ‘coin, big, tennis’).

#### 3.2.8. Demographic questionnaire

Lastly, a demographic questionnaire was administered to all participants. In this questionnaire, only age and gender were assessed.

### 3.3. Procedure

The procedures of the proposed replications were executed as closely as possible to that of Heintzelman *et al*. [11]. As such, like the original study, eligible participants were compensated $1.00 for their participation in either preregistered replication study. Participants were recruited for this replication online via Amazon's Mechanical Turk (MTurk) system. Parameters on MTurk were set such that participants were only allowed to preview the study if they (i) were located in the USA, (ii) had a lifetime approval rating of greater than or equal to 95%, and (iii) had at least one prior work approval. Participants having already completed one of the three prior unregistered conceptual replication studies were not allowed to participate in either of the present preregistered replications. Further, participants having completed one of the preregistered replications were not allowed to complete the other. As such, both studies comprised unique participants.

For the replication of Heintzelman *et al*.'s [11] Study 2, participants were first presented with a consent form approved by the present authors' Institutional Review Board (IRB). After agreeing to participate in the present research, participants were randomly sorted into one of three conditions: seasonal (spring, summer, autumn, winter), arbitrary (e.g. summer, autumn, spring, winter, summer, autumn, spring, winter, etc.) or random ordering of tree pictures. To control for possible order effects, participants were counterbalanced by randomly assigning them further to one of eight intracondition photo orders. As such, any given participant was sorted into 1 of 24 possible photo presentations. Each condition/order, however, showed the same 16 unique photos of trees in seasonal change. With each photo, participants were asked to (i) rate the contrast of the photo, and (ii) select the colour most frequently occurring in the photo from an array of colour swatches. Time spent on each page was covertly monitored, and participants were allowed to move on to the next photo at their own discretion. After participants viewed their 16 photos, participants completed the MIL, EPA/ENA and IPA/INA measures in a counterbalanced order. Within the MIL, an attention check asking participants to select a specific answer category was present. After completing the three measures, participants completed the optional pattern check and the demographics questionnaire. Finally, participants were given a verification code for MTurk to confirm their participation and receive compensation.

For the replication of Heintzelman *et al*.'s [11] Study 4, participants first viewed its respective IRB-approved consent form. After agreeing to participate, participants were randomly assigned to view coherent or incoherent linguistic triads (cf. [16]). During the viewing of the linguistic triads, nothing else was on the survey page except for the survey header and one linguistic triad. Triads in both conditions automatically advanced to the next triad after 4 s of exposure. All participants viewed a total of 10 distinct linguistic triads during this portion of the experiment. Following the presentation of the linguistic triads, all participants completed the MLQ and the EPA/ENA measures in a counterbalanced order. The MLQ also included an attention check, as outlined above. Participants ended the survey with the demographics questionnaire, and receipt of their MTurk verification code for successful completion of the study.

### 3.4. Analytic strategy

A replication of both the unadjusted and the adjusted effects of Study 2 and Study 4 in Heintzelman *et al*. [11] will be attempted. After data collection, but before any actual analysis, data will be screened for missing values and outliers. Following the strategy employed by Heintzelman *et al*. [11], individuals who do not answer any questions in a given section will be excluded from analyses which use the measure in question. Individuals with partially observed data (e.g. individuals who skipped a question) will also be retained on an analysis-by-analysis basis. In other words, if an individual has complete data on all variables that were used in an analysis, this participant will be retained. Otherwise, this participant is to be excluded from analysis.

In the interest of having an explicit preliminary plan for this replication, two slight deviations from the original study will be made in that predetermined exclusionary criteria for data abnormalities will be in place. First, individuals who are marked as outliers in univariate boxplots on outcome measures (using the 2.5 times interquartile range criterion) will be discarded from analyses. Second, an attention check will also be included in the survey (a question that asks respondents to check a particular answer category). Individuals who fail this attention check will also be discarded. The underlying assumption is that these individuals are probably not filling out the survey truthfully, but only in order to get the monetary reward.

After these data screening and cleaning, the exact same statistical quantities as in the original studies will be computed. In particular, the following Frequentist quantities for the replication of Heintzelman *et al*.'s [11] Study 2 will be estimated:

(1) An *unadjusted* effect comparing the mean of perceived MIL in the aggregate of the seasonal and arbitrary condition with the mean of the same variable for the group in the random condition. This will be estimated using a simple contrast coefficient. In particular, the categorical variable will be recoded into three levels of two contrast codes: one comparing the average of the seasonal and arbitrary condition against the random condition, and an orthogonal contrast that compares the seasonal and arbitrary condition. This second contrast is not of substantive interest, but it ensures that the standard error is computed across all three cells of the design. In fact, only this particular contrast coding scheme ensures that inferential statistics are identical to using analysis of variance (ANOVA) and a custom contrast. The inferential test of the first contrast coded variable represents the central effect to be replicated in Study 2. Because affect measures are not central to the effect in question (and with the original study finding no effect), groups will not be compared on these or any other additional outcomes.
(2) An *adjusted* effect comparing the mean of MIL in the aggregate of the seasonal and arbitrary conditions with the mean of the same variable for the group in the random condition. This will be estimated using linear regression. Just as in the original paper, the adjustment variables will be the time spent on each photo, EPA, ENA, IPA and INA.

For the replication of Heintzelman *et al*.'s [11] Study 4, the following effects will be estimated:

(1) An *unadjusted* effect comparing the mean of MIL between the two groups that received incoherent or coherent word triads will be conducted. This is the central effect to be replicated in Study 4 and will be estimated using an independent samples *t*-test. Again, affective measures are not central to our replication and coherent and incoherent groups will not be compared on these or any other additional outcomes.
(2) An *adjusted* effect comparing the mean of MIL between the two conditions, using linear regression will be performed. Just as in the original paper, the adjustment variables will be EPA and ENA.

In addition to the Frequentist analyses above that mirror the ones conducted in the original study, Bayesian versions of all analyses will also be performed. In particular, for the replication of Heintzelman *et al*.'s [11] Study 2, the following quantities will be computed:

(1) A Bayes factor for the critical comparison of the combined means of MIL of the two coherent groups versus the mean of the incoherent group will be generated. To mirror the Frequentist analysis, a Bayes factor that compares a full model (one with both contrast codes) to a model that omits the contrast code that represents the focal hypothesis will be performed. A Jeffreys–Zellner–Siow (JZS) prior (as suggested by Rouder *et al*. [26]) with a suggested medium width of 0.707 of the Cauchy distributed prior will be used. This particular prior is centred around zero (thus not favouring an effect in either direction away from the null) and it is still wide enough to accommodate potentially large effects in either direction.
(2) A Bayes factor that compares a regression model with the same outcome and all predictor variables mentioned above in the Frequentist analysis and a regression model that is identical, but omits the critical predictor of treatment condition will be computed. The same prior distribution discussed above will be used here as well.

For the replication of Heintzelman *et al*.'s [11] Study 4, the following Bayesian analyses will be performed:

(1) A Bayes factor that compares a null model (i.e. a model with no predictors) against a model that includes the treatment assignment as a predictor of MIL will be conducted. As a note, this is identical to a Bayesian *t*-test that compares a null model in which the mean differences are zero versus a model in which mean differences may be non-zero. Again, the JZS prior will be employed.
(2) A Bayes factor that again compares a regression model that predicts MIL from all variables mentioned in the Frequentist analogue above versus a model that includes the same variable, but omits the treatment assignment predictor will be created. The JZS prior will again be used here.

No other preregistered data analyses will be conducted, and no other statistical significance test on any other outcome will be computed. Therefore, tests of each of the four effects will use an unadjusted 0.05 significance level (just as the original study did). Should any additional analyses be conducted, they will explicitly be labelled as separate from the approved protocol and therefore exploratory in nature. This study was submitted to the Open Science Framework (OSF) after gaining provisional acceptance from the journal, and gained preregistered status on 5 July 2016. Analysis code was written and submitted prior to data collection, and is available alongside the raw data and relevant study materials at the authors' preregistered repository (see https://osf.io/t4jsq/). In addition to the authors' repository, electronic supplementary materials for this submission are also available online at rs.figshare.com.

## 4. Results

### 4.1. Replication of Heintzelman *et al*. [11] Study 2: trees in seasonal change

#### 4.1.1. Data cleaning

Of the variables relevant to this study's analyses, checks for missingness revealed no missing values on any predictor, covariate or outcome. A check of the univariate boxplot for the outcome measure, MIL, revealed no outlying data points. Finally, it was found that four individuals failed the attention check. In accordance with our preregistered procedures, these individuals were excluded from all study analyses. Our final sample for the preregistered analyses therefore contained 478 valid observations. Descriptive statistics (for both aggregate values, and values split according to condition) can be found in table 1.

**Table 1.** Descriptive statistics for Study 2 replication, aggregate and by experimental condition. MIL, meaning in life; EPA, explicit positive affect; ENA, explicit negative affect; IPA, implicit positive affect; INA, implicit negative affect.

	total (*N* = 478)	seasonal (*n* = 157)	arbitrary (*n* = 161)	random (*n* = 160)
study variables	*M* (s.d.)	*M* (s.d.)	*M* (s.d.)	*M* (s.d.)
MIL	4.71 (1.66)	4.80 (1.54)	4.68 (1.69)	4.64 (1.76)
EPA	4.48 (1.59)	4.51 (1.54)	4.36 (1.61)	4.57 (1.63)
ENA	2.24 (1.51)	2.28 (1.54)	2.13 (1.49)	2.32 (1.51)
IPA	3.09 (1.26)	3.09 (1.29)	3.00 (1.18)	3.18 (1.29)
INA	2.52 (1.02)	2.54 (1.02)	2.45 (0.96)	2.59 (1.08)
time (sum)	229.6 (154.21)	223.61 (126.34)	221.04 (130.93)	244.10 (195.22)

Diagnostic plots were generated to check the assumptions of the impending linear models to test our effects of interest. It is important to note, diagnostic plots of the residual values of Study 2's adjusted linear model showed evidence that suggested some model misspecification. As data probing for potential model misspecification did not appear to be a part of the original procedure, it was not preregistered as a part of this direct replication. To explore this potential misspecification, we performed a relatively exhaustive probing of the data. Our best solutions and conclusions can be found in the ‘Unregistered exploratory analyses’ section that follows our preregistered analyses.

### 4.2. Preregistered analyses: unadjusted and adjusted effects

#### 4.2.1. Frequentist

In preparing to analyse the unadjusted effect for condition on MIL, a Levene's test of the homogeneity of variances was conducted to verify the assumptions of the general linear model. The test suggested that the variance for MIL among the three condition categories did not significantly vary, thus, fulfilling this critical assumption (*p* = 0.088). Moving forward, the overall one-way unadjusted ANOVA found that MIL did not significantly vary by condition, *F*_2,475_ = 0.35, *p* = 0.705, *η*^2^ = 0.001. To test our critical comparison of MIL in coherent (average of seasonal and arbitrary) versus incoherent (random) conditions, a contrast code was applied to appropriately weigh the groups. Again, the assumption of homogeneity of variance was checked by way of a Levene's test for the contrast-applied model, and found to be fulfilled (*p* = 0.110). The resultant mean difference for MIL between coherent and incoherent groups also failed to reach significance, *t*_476_ = 0.59, *p* = 0.554, 95% CI [−0.22, 0.41]. The estimated effect size for this contrasted model was modest, *d* = 0.06.

To test the adjusted effect of condition on perceived MIL, a linear regression was constructed with EPA, ENA, IPA, INA and Time entered as covariates. A contrast code was then applied to the regression model to test coherent (average of the seasonal and arbitrary) versus incoherent (random) conditions. Although the overall model of the regression was indeed significant (*F*_7,470_ = 23.17, *p* < 0.001; *R*^2^ = 0.26, adjusted *R*^2^ = 0.25), condition $\left(\eta_{p}^{2}=0.002\right)$ failed to emerge as a significant predictor of MIL when entered alongside the listed covariates. Coefficient estimates for this adjusted regression can be found in table 3.

#### 4.2.2. Bayesian

As proposed in our protocol, Bayesian analyses were also conducted to mirror the Frequentist analyses found above. First, a general Bayes factor was calculated using the JZS prior to test a model where experimental condition predicts MIL against a null model (using the *BayesFactor* package in R [27]). The Bayes factor yielded here was found to be BF_01_ = 0.033. As such, the data show strong evidence for the null model. After manually applying our contrast statement to test our focal hypothesis regarding the unadjusted effect of coherent (average of seasonal and arbitrary) versus incoherent (random) conditions on MIL against a null model, the analysis yielded BF_01_ = 0.185, suggesting moderate evidence for the null model.

Finally, to test the adjusted effect, we computed a Bayes factor comparing a model using the contrast-weighted condition variables alongside EPA, ENA, IPA, INA and Time predicting MIL, to a model that omits the contrast-weighted condition containing our focal effect as a predictor. This analysis yielded BF_01_ = 0.285, again indicating moderate evidence in favour of the model that does not include our weighted condition contrast (average of the coherent, cyclical groups versus the incoherent, random group) as a predictor of MIL. In other words, this yields evidence that the null hypothesis of the condition effect being zero was moderately supported. In summary, all Bayesian analyses are congruent with the findings of the Frequentist analyses above. The Bayesian analyses allow quantification of support of the null hypothesis over a specified alternative, and all models indicated moderate to strong support for the null hypothesis.

### 4.3. Replication of Heintzelman *et al*. [11] Study 4: linguistic triads

#### 4.3.1. Data cleaning

Checks for missing data in Study 4 revealed complete data for all relevant study variables. Furthermore, no cases appeared as outliers on the univariate boxplot check of the outcome variable, MIL. A total of three individuals appeared to fail the attention check, and in accordance with our study protocol, these cases were subsequently eliminated from the dataset. The final sample comprised 291 valid observations. Descriptive statistics can be found in table 2 for variables relevant to Study 4. Diagnostic plots of the following preregistered analyses showed no concerning evidence of model misspecification or violations of linear assumptions.

**Table 2.** Descriptive statistics for Study 4 replication, aggregate and by experimental condition. MLQ-P, Meaning in Life Questionnaire, Presence subscale [25]; EPA, explicit positive affect; ENA, explicit negative affect.

	total (*N* = 291)	coherent (*n* = 144)	incoherent(*n* = 147)
study variables	*M* (s.d.)	*M* (s.d.)	*M* (s.d.)
MLQ-P	4.76 (1.54)	4.68 (1.64)	4.83 (1.44)
EPA	4.33 (1.51)	4.22 (1.50)	4.44 (1.52)
ENA	2.73 (1.66)	2.83 (1.75)	2.64 (1.57)

### 4.4. Preregistered analyses: unadjusted and adjusted effects

#### 4.4.1. Frequentist

Per the preregistered protocol, the first test in the Study 4 sequence will be an independent samples *t*-test investigating the unadjusted comparison of coherent and incoherent groups with regard to perceived MIL scores as assessed by the MLQ-P [25]. A Levene's test of equality of variances was not significant. The regular independent samples *t*-test failed to reach the predetermined threshold for significance, *t*_289_ = −0.845, *p* = 0.399, 95% CI [−0.51, 0.20], *d* = 0.099.

To test the adjusted effect of interest, linear regression was again used to predict perceived MIL from experimental condition plus covariates, EPA and ENA. While the overall model was again significant (*F*_3,287_ = 46.62, *p* < 0.001; *R*^2^ = 0.33, adjusted *R*^2^ = 0.32), experimental condition $\left(\eta_{p}^{2}=0.00004\right)$ did not significantly predict MIL scores above and beyond covariates, EPA and ENA. Coefficient estimates for this adjusted regression can be found also in table 3.

**Table 3.** Coefficient estimates for predicting MIL: adjusted effects. In both studies, coefficients should be interpreted as movement from coherent (average of seasonal and arbitrary patterns in Study 2; coherent linguistic triads in Study 4) to incoherent group (random group in Study 2; incoherent linguistic triads in Study 4). EPA, explicit positive affect; ENA, explicit negative affect; IPA, implicit positive affect; INA, implicit negative affect.

predictor variables	*B* (s.e.)	*T*	*P*	95% CI (upper, lower)
Study 2: tree photos				
condition	0.14 (0.14)	1.03	0.305	[−0.13, 0.42]
EPA	0.43 (0.05)	8.90	<0.001	[0.34, 0.53]
ENA	−0.17 (0.05)	−3.41	0.001	[−0.27, −0.07]
IPA	0.07 (0.06)	1.18	0.240	[−0.05, 0.20]
INA	0.01 (0.08)	0.09	0.926	[−0.14, 0.16]
time (sum)	−0.0002 (0.0004)	−0.58	0.562	[−0.001, 0.001]
Study 4: linguistic triads				
condition	0.02 (0.15)	0.11	0.913	[−0.28, 0.31]
EPA	0.56 (0.05)	10.48	<0.001	[0.45, 0.66]
ENA	−0.06 (0.05)	−1.32	0.189	[−0.15, 0.03]

#### 4.4.2. Bayesian

A Bayesian analysis was employed to compare with and extend the Frequentist tests outlined previously. To test the unadjusted effect of interest, a Bayes factor was computed using the JZS prior for a model testing experimental condition as a predictor of MIL scores versus a null model. BF_01_ was 0.181, suggesting moderate evidence in favour of the null model.

Finally, to test the adjusted effect of interest under Bayesian methodology, a Bayes factor was computed using the JZS prior to test a model predicting MIL scores from experimental condition, EPA and ENA versus a model with experimental condition omitted from the list of predictors (i.e. MIL scores being predicted by EPA and ENA alone). BF_01_ in this test was 0.135, suggesting again moderate evidence in favour of the denominator, or in this case, the model with MIL scores being predicted from EPA and ENA alone. In other words, there was moderate evidence that the null hypothesis of no effect of the treatment was supported.

### 4.5. Unregistered exploratory analyses

In our preregistration of the analyses, we described using the sum of all times that participants look at the stimuli as a covariate. However, it later occurred to us that the original authors probably used average time (across all stimuli). Using a mean instead of a sum is a simple linear transformation (sums divided by number of stimuli), and therefore does not have any effect on our analyses. Nevertheless, we reran our models with the average time, which, as expected did not change our results (except the time coefficient which was rescaled, but retained the same inferential statistics).^1^

**Figure 1.**
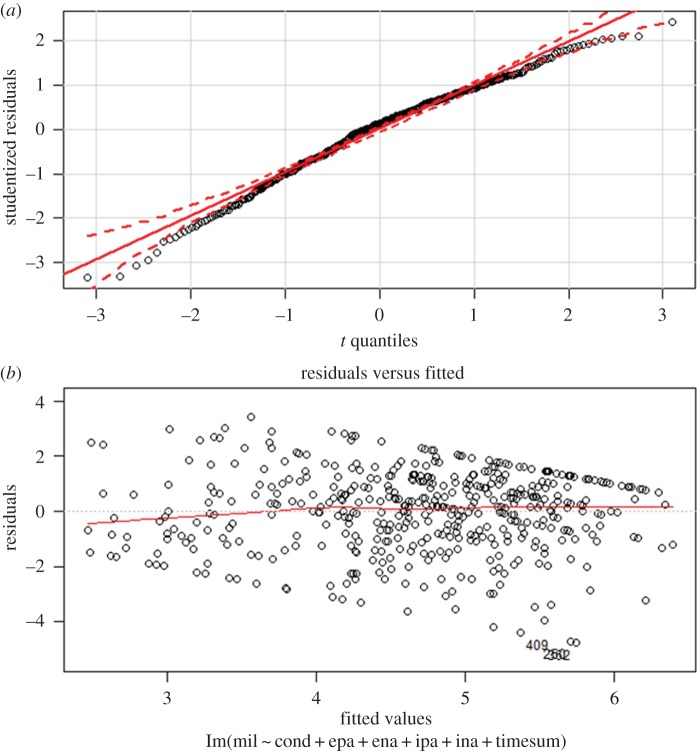
(*a*) QQ-plot of the residuals of the adjusted effect of Study 2 against quantiles. Deviation from the 45° diagonal (solid line) suggests model misspecification, most commonly, skewness. Many data points here fall outside of the 95% confidence interval (dotted lines). (*b*) Residuals versus fitted plot of the adjusted effect of Study 2. Deviation from the constant line again suggests slight model misspecification.

Second, as foreshadowed in our data cleaning section, we noted that the regression model in Study 2 that included all covariates showed slight, but notable patterns in the residuals. A QQ-plot (figure 1*a*) suggested slight non-normality of the residuals, and a fitted versus residual plot suggested a very slight deviation from a straight line (figure 1*b*). To explore the source of this potential violation of regression assumptions, we tried two strategies. First, we fitted a model that included all interactive terms between treatment assignment and the covariates, and all quadratic terms of all covariates. We therefore created a model in which linearity and additive assumptions were related. This model, however, did not yield much better looking residuals. Our second strategy was to perform a Box–Cox transformation of the outcome variable in the full regression. The Box–Cox procedure suggested powering the outcome variable by 1.5. After this transformation, all relationships appeared more linearized, as evidenced by much better looking residuals. The overall model was again found to be significant (*F*_6,449_ = 25.19, *p* < 0.001; *R*^2^ = 0.25); however, the weak relationship between contrast-weighted experimental condition and the outcome remained (*B* = 0.24, s.e. = 0.44, *t* = 0.56, *p* = 0.579). Given that powering the outcome by 1.5 does not yield immediately interpretable scores, it suffices to say that even linearizing relationships did not alter results.

**Figure 2.**
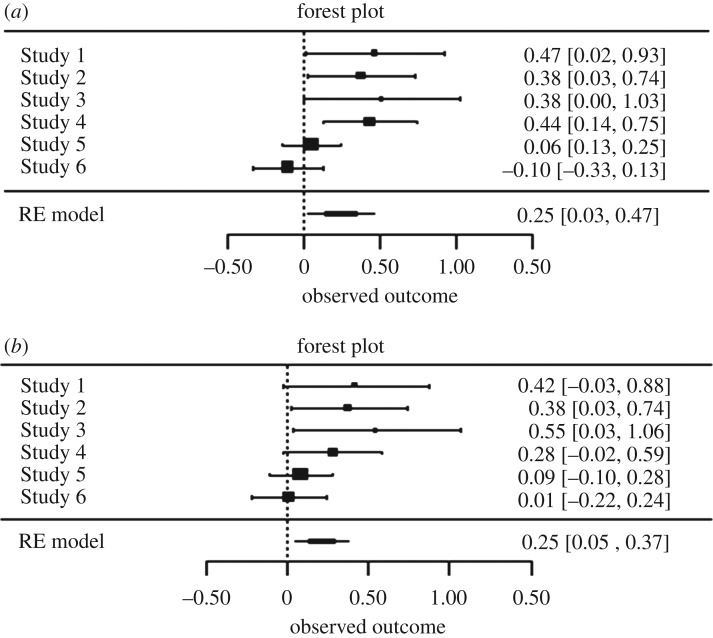
Forest plots of the (*a*) unadjusted and (*b*) adjusted effect sizes for the original paper (Studies 1–4) and the present replication attempts (Studies 5 and 6 corresponding to replications of Studies 2 and 4 of the original paper, respectively).

Finally, as suggested by Braver *et al*. [28], it is often beneficial to supplement replication attempts with meta-analytic effect sizes estimates—even if the number of studies is low. Because all of the reported studies of Heintzelman *et al*. [11] examined the same effect (albeit using different stimuli), we decided to use effect size estimates from all four studies of the original paper. We collected both unadjusted and adjusted effects for a total of eight effect sizes, all comparing a random and a coherent pattern. For each collected effect size, we recorded which stimuli were used. When available, reported summary statistics were used to compute Cohen's *d* standardized effect sizes. When reported effects averaged over two experimental groups, we formed weighted averages of means and variances and computed Cohen's *d* from these statistics. When effects were reported in regression models, adjusted means from these regression models and original standard deviations from sample descriptive statistics were used to form Cohen's *d* effect sizes. In addition to the effect sizes from the original paper, we also added all effect sizes from this replication study to the meta-analysis. Using the *metafor* package in R [29], a random-effects meta-analysis was conducted. Unadjusted and adjusted effects were analysed separately, because these effects are not independent of one another. The meta-analysis of the four unadjusted effect sizes from the original paper, and the two replicated effect sizes, yielded an overall standardized effect of 0.246, *z* = 2.20, *p* = 0.028, 95% CI [0.027, 0.465], indicating a significant effect (figure 2*a*). The observed heterogeneity was *Q*(5) = 14.49, *p* = 0.013, indicating significant heterogeneity. This heterogeneity, however, was attributable to being part of the set of original or replication effect sizes. Including replication status as a moderator in the meta-analysis, revealed significant differences in effect sizes, *b* = −0.447, *z* = −3.64, *p* = 0.0003. The residual heterogeneity was very low, *Q*(4) = 1.26, *p* = 0.869. Type of stimulus was not a significant moderator of the meta-analytic effect size, *b* = −0.030, *z* = −0.12, *p* = 0.903. The analysis of adjusted effect sizes yielded very comparable results. The overall meta-analytic effect size estimate was 0.214, *z* = 2.63, *p* = 0.009, 95% CI [0.054, 0.373], again indicating a significant overall effect (figure 2*b*). Overall, heterogeneity was not significant, *Q*(5) = 7.78, *p* = 0.169, yet status of either original or replication was again a significant moderator of the meta-analytic effect size, *b* = −0.318, *z* = −2.59, *p* = 0.010. All R code for these unregistered exploratory analyses can be found in our data repository on OSF.

## 5. Discussion

Recognizing the unique and valuable implications of Heintzelman *et al*. [11], we sought to directly replicate these effects of exposure to objective coherence on perceived MIL. If presentation of coherent/incoherent stimuli could indeed change one's level of felt MIL, the field would not only advance towards understanding how individuals find MIL, but it would also point to future directions for researchers interested in helping individuals achieve and maintain this prominent existential task. Our replication of Heintzelman *et al*.'s [11] Study 2 tested if perceived MIL significantly differed between groups exposed to either a coherent (an average of seasonally ordered and non-seasonally ordered, but repeating, ‘arbitrary’ patterns) or incoherent (randomly ordered) pattern of tree photographs. The results of the present attempt failed to replicate the original findings of Heintzelman *et al*. [11] in that perceived MIL did not appear to statistically differ between the coherent and incoherent groups following exposure to their respective stimuli. Even after controlling for several known correlates of perceived MIL (e.g. [16,30,31]), experimental condition did not emerge as a significant predictor above and beyond these covariates in Study 2. In our replication of Heintzelman *et al*.'s [11] Study 4, participants were exposed to coherent and incoherent linguistic triads to examine whether these stimuli could differentially impact perceived MIL. In our attempt to replicate, perceived MIL scores did not significantly differ by experimental condition. Again, after adjusting for known correlates of MIL, experimental condition fell short of reaching the threshold for statistical significance. As such, we are left with several discrepancies in this attempt to replicate the findings of Study 2 and Study 4 in Heintzelmen *et al*. [11].

Following suggestions put forth by Braver *et al*. [28] for replication attempts, exploratory analyses of the meta-analytic effect size estimates were initiated. Both unadjusted and adjusted effects were computed, resulting in two separate meta-analytic effect size estimates each comprising four studies from the original paper and two replications from this study. For both unadjusted and adjusted meta-analytic effect size estimates, the six pooled studies indicated an effect size significantly different from zero thus suggesting rejection of the null hypothesis (i.e. that there is no effect of objective coherence on perceived meaning). Two important caveats, however, should be noted. First, both unadjusted and adjusted meta-analytic effect size estimates were significantly moderated by status as an effect of the original study or replication attempt. Second, the pooling of the six effect sizes in both meta-analyses resulted in two aggregate effect sizes that were significant, but markedly attenuated relative to the initial studies. To be sure, it is clear from the reviewed analyses and apparent discrepancies that further adjudication of the effect in question will be necessary.

Prior to discussing these results further, we feel strongly the need to caution readers against the unequal weighing of our replication attempt against the original Heintzelman *et al*. [11] findings. An unsuccessful replication attempt may—at best—demonstrate that a given effect is unreliable. Although we do not wish to undermine the importance of replication in helping fields move towards more accurate understandings of phenomena, we want to be unequivocal in our stance that a single unsuccessful replication attempt *does not* invalidate the findings of the original study, nor does it disprove that an effect exists (see also [32]). In this study, we have taken careful steps to justly test our effects of interest by adequately powering our studies, and approaching our research questions from both Frequentist and Bayesian schools of analysis. Our diligence, however, does not change the fact that the present study is but one replication attempt, and prior researchers (e.g. [33]) have asserted that multiple attempts at replication are often required to ascertain true effects.

In addition to future direct and conceptual replications, several avenues for expansion are possible for this effect of exposure to objective coherence on perceived MIL. First, as put forth by Heintzelman *et al*. [11],^2^ we agree that any expansion of the present studies would greatly benefit from the inclusion of a control group. At the present time, it cannot be determined if the original findings of Heintzelman and her colleagues were the result of an increase in perceived meaning in the coherent groups, or a decrease in perceived meaning in the incoherent groups. Second, as indicated in our study procedures, the present investigation deviated in one way from the original Heintzelman *et al*. studies with the inclusion of an explicit attention check embedded in an outcome measure following stimuli presentation. Although attention checks have become commonplace in, recommended for, and found to generally improve the quality of data obtained from crowdsourcing recruitment methods such as MTurk [34–36], its inclusion is indeed an important difference between the original paper and our replications. Research has suggested that ‘tricky’ attention checks [37] may have a significant impact on subsequent responses [38]. To the best of our knowledge, no study has tested the effects of explicit attention checks (e.g. asking participants to mark a certain response, as we did in this study). It is an empirical question for future investigations if presumably overt and neutral attention checks may significantly impact participant responses or moderate certain results. Third, the issue with the reconciliation of Heintzelman *et al*.'s findings with prior MMM-related research [17] would benefit from further investigation. Although one might have expected that the incoherent groups of Heintzelman *et al*.'s study would experience an increase in perceived meaning following Heine *et al*.'s [17] concept of fluid compensation, to our knowledge, no study has investigated the importance of time between exposure to a meaning threat and fluid compensation. Perhaps it is the case that some amount of ‘lag’ occurs before the installation of fluid compensation. As such, it is possible that one may observe a temporary change in meaning (e.g. a decrease in perceived meaning, as may be in the case of [11]) or no change at all (as may be in the case of our present replication attempt) for a very brief period following exposure to a meaning threat before fluid compensation is fully activated. On this note, the foundational building blocks of the effects in question (e.g. the MMM) would benefit from future investigation and replication themselves. In the light of the issues facing psychology with regard to replication, it is important that researchers avoid developing ‘tunnel vision’ when investigating particular effects. Instead, a bottom-up approach to the re-examination of our theoretical paradigms might behove the field.

Finally, it is important to highlight that not all of the findings in the present studies failed to reach indicated thresholds for significance. Rather, a reliable correlation between explicit affect and perceived MIL was demonstrated. Replicated across both of the present studies, it appears that individuals who report more positive affect also tend to report higher feelings of perceived MIL. This finding is consistent with several prior works on the effects of mood on feeling as though one's existence is meaningful (e.g. [16,30,39]). It is hoped that our replications of this effect adds to the literature solidifying affect as having a large role in one's attainment of meaning.

Preregistered replication attempts are but one of the many methods researchers in the social sciences are using to help bolster confidence in emerging findings. With regard to the research being conducted on ethereal topics such as fate, meaning and purpose, there is a long but promising road ahead towards understanding these constructs and what gives rise to them in the course of everyday life. The failure of coherence to elicit a greater sense of meaning in the current study does little to explicate what does, in fact, elicit such a sense. There is a growing body of the literature to support the effects of structure in the environment on perceived meaningfulness and related processes (e.g. [40–42]); however, in the light of the present findings, researchers may wish to revisit the theorized pathways by which perceived coherence in external stimuli should promote greater meaning and consider potential boundary conditions that may contour this process. The process by which we establish MIL, in particular, probably comprises many mechanisms working together in concert. Although it was not an explicit end goal of the original paper, every small step towards illuminating this process serves as another step towards researchers discovering effective ways to help all individuals secure this elusive, yet coveted, sense and work their way towards psychological flourishing.

## Supplementary Material

R Studio Script

## Supplementary Material

Study 2 raw data (CSV files) from The effects of exposure to objective coherence on perceived meaning in life: a preregistered direct replication of Heintzelman, Trent & King (2013)

## Supplementary Material

Study 4 raw data (CSV files) from The effects of exposure to objective coherence on perceived meaning in life: a preregistered direct replication of Heintzelman, Trent & King (2013)

## Footnotes

1 Like the preregistered analysis using the summed total time, the overall model of the regression using the average of time spent on photographs was significant (*F*_7,470_ = 22.12, *p* < 0.001; *R*^2^ = 0.25, adjusted *R*^2^ = 0.24). Condition (*B*[s.e.] = 0.14[0.11], *t* = 1.21, *p* = 0.225; 95% CI [−0.09, 0.36]), however, failed to emerge as a significant predictor of MIL when entered alongside explicit positive affect (*B*[s.e.] = 0.35[0.04], *t* = 8.76, *p* < 0.001; 95% CI [0.27, 0.42]), explicit negative affect (*B*[s.e.] = −0.13[0.04], *t* = −3.35, *p* = 0.001; 95% CI [−0.21, −0.05]), implicit positive affect (*B*[s.e.] = 0.06[0.05], *t* = 1.14, *p* = 0.254; 95% CI [−0.04, 0.16]), implicit negative affect (*B*[s.e.] = 0.001[0.06], *t* = −0.02, *p* = 0.981; 95% CI [−0.12, 0.12]), and the average time spent on each photo (*B*[s.e.] = −0.002[0.01], *t* = −0.36, *p* = 0.717; 95% CI [−0.01, 0.01]).

2 This point was also suggested by a reviewer for Stage 1 of the present article. We elected not to include a control group at this time because the explicit intent of the present studies were to directly replicate Heintzelman *et al.* [11]. Further, the inclusion of a control group would have taxed our resources significantly by requiring us to increase our sample size in order to attain sufficient power for replication.

## Data accessibility

Following manuscript approval, this study was preregistered with the OSF (https://osf.io/pzf2e/). Following successful preregistration of our study, data collection began. All raw data, stimuli, R code and associated study materials are available at our study's initial project repository: https://osf.io/t4jsq/.
